# Flap-necked chameleons change colour to match their background

**DOI:** 10.1098/rsbl.2025.0134

**Published:** 2025-08-06

**Authors:** Tom Major, Alexia C. M. Hesten, Jan Stipala, Michael A. Cant, Martin Stevens, Jolyon Troscianko

**Affiliations:** ^1^ Centre for Ecology and Conservation, University of Exeter, Penryn, UK; ^2^ Department of Life & Environmental Sciences, Bournemouth University, Poole, UK; ^3^ School of Biological and Environmental Sciences, Liverpool John Moores University, Liverpool, UK

**Keywords:** colour change, crypsis, chameleon, social signalling, camouflage

## Abstract

Popular culture leads us to believe that chameleons change colour to match their backgrounds, yet surprisingly, this ability has rarely been demonstrated under controlled conditions. Existing research shows that colour change is primarily used for social signalling and thermoregulation, and that chameleons revert to a generic background-matching colour for camouflage rather than tuning their colour to specific backgrounds. Here, to test the background-matching abilities of flap-necked chameleons (*Chamaeleo dilepis*), we placed them on backgrounds of various standardized colours and patterns and measured their appearance over time using models of predator vision. We found that chameleons could change their brightness to better match black backgrounds and change colour to match some hues, especially yellow. They did not match background patterns. In demonstrating that flap-necked chameleons use colour and brightness changes to facilitate camouflage, we provide further evidence supporting this function of colour change in chameleons.

## 1. Introduction

Chameleons are renowned for their ability to change colour and are often portrayed in popular culture as being able to take on the colour of their environment for concealment. However, research has shown that their colour change is governed by a range of ecological and behavioural factors. Colour change can broadly be separated into physiological and morphological colour change, the former being relatively quick and often involving changes in the state (e.g. dispersion or aggregation) of pigment within chromatophore cells, whereas the latter is slower and often involves more fundamental changes in tissue structure and chromatophore cell distributions or numbers [1]. The primary function of colour change in chameleons is thought to be conspecific communication [1–5], and chameleons are well known to change colour during the breeding season to scare off rivals and signal to potential mates whether they wish to copulate [6,7]. Colour changes also provide thermoregulatory benefits [8–11].

The third proposed function of chameleon colour change is camouflage to match their background. Background-matching colour change is known to be highly context specific and affected by a variety of ecological factors [12]. Rapid physiological colour change for concealment (facultative crypsis) has been investigated as a response to a perceived predatory threat from snakes and birds, with dwarf chameleons altering their appearance based on perceived risk [3,4]. Despite this research, colour change for concealment is surprisingly poorly understood in chameleons, and previous studies have focused on whether camouflage is ‘switched on’ when a predator appears. Considering the life history of a chameleon as a slow but mobile predator under constant threat from its own predators [13], it seems logical that colour and pattern changes be used to remain unnoticed by potential threats whenever possible.

Colour change is known to be context specific in chameleons, with veiled chameleons (*Chamaeleo calyptratus*) more likely to rely on colour change in closed than open habitats [14]. Additionally, individuals who possess a superior ability to match their surroundings in the context of a bird visual model are also more likely to use it as an antipredator defence [14]. Colour-changing abilities can vary between populations. For example, invasive Jackson’s chameleons (*Trioceros jacksonii*) in Hawaii, free from the threat of predators with good colour discrimination, demonstrate reduced colour change as an antipredator response and more conspicuous display coloration to conspecifics, compared with the Kenyan source population [15]. In common chameleons (*Chamaeleo chamaeleon*), small, subordinate ‘sneaker’ males switch to courtship displays more quickly than larger males and are less likely to adopt crypsis in the presence of a female [7]. By contrast, larger males prefer to guard their mates and adopt the colour of the background [7].

Despite the above work on chameleon colour change, the extent to which chameleons change their appearance for camouflage remains unclear. Beyond a limited number of controlled tests (e.g. [14]), there remains a lack of research into the colour and pattern matching abilities of chameleons, including changes in the absence of predators (which is important as predator visual systems are likely to have longer detection distances than their prey) and in the time taken to achieve effective colour change. Common chameleons (*C. chamaeleon*) have been shown to switch between brown and green coloration when placed on artificial bushes of those colours [7], but do not change to match off-white, yellow or blue bushes. However, these changes were not measured over time, but the speed of change is of ecological relevance because physiological colour change can take several minutes to occur and provides insight into how the animal uses its colour-changing ability [1]. Similarly, luminance and hue changes were not measured separately or using models of predator vision. As different hues can have different luminance, separating out these measures during testing is important to get an accurate understanding of what prompts colour change. Furthermore, anti-predator camouflage abilities will have likely evolved in response to the visual abilities of an animal’s predators, so assessing colour change from a suitable predator’s visual system is important [16]. In this case, we used the peafowl model as this was the closest one available to a key predator of chameleons, fiscal shrikes (*Lanius collaris*). The background-matching abilities of dwarf chameleons (*Bradypodion taenibronchum*) have previously been demonstrated in the wild, using models of predator vision to compare chameleons to the colour of perches and leaves [3,4,7]. While effective, the outdoor environment precludes the ability to control temperature, lighting or other factors that might influence colour change. Both dwarf chameleons and common chameleons are capable of quickly changing the colour of body patches to present a contrasting, patterned appearance for social signalling [7,17]. If chameleons possess this ability, their camouflage might also involve matching the pattern of their surroundings rather than (or in addition to) hue, but this has not been investigated.

It is commonly accepted that flap-necked chameleons (*Chamaeleo dilepis*) have the capacity to rapidly change colour and pattern [18]. Here, we investigated the camouflage response of flap-necked chameleons to artificial environments of varying (i) hue, (ii) brightness, and (iii) pattern, while other environmental variables were controlled. We measured colour change using animal vision modelling and non-intrusive methods to reduce stress-related colour change [3].

## 2. Material and methods

### (a) Data collection

To test their colour-changing capabilities, chameleons were exposed to four uniform colour experiments (yellow, yellow-green, orange and blue-green), three uniform greyscale experiments (black, white and grey) and six pattern experiments. Colour treatments were uniform hues of consistent luminance (electronic supplementary material, figure S1). We selected hues that were slightly more colourful than typical natural backgrounds to elicit a stronger response from the chameleons, while still preserving biological plausibility. Yellow-green and blue-green colours were selected as colours which other chameleons are able to produce [19], and orange and yellow could be of ecological relevance to flap-necked chameleons that experience pronounced dry seasons, where leaves and grasses may change to these colours [20]. Pattern treatments were either yellow and yellow–green, or black and white, at three different spatial scales, constituting patterns consisting of small, medium or large patches (see electronic supplementary material for further details of pattern creation, figure S2). Individual chameleons were placed into a 30 × 90 × 30 cm experimental arena constructed of corrugated plastic (corex) with a square perch in the centre. We initially exposed each individual to 20 min in a control arena of uniform grey walls, floor and perch to standardize any residual patterning or colour from their cages, during which time chameleons were photographed immediately and then after 1, 5, 10 and 20 min. The chameleon was then moved into a second experimental arena in which the walls, floor and perch were covered in paper with the uniform colour or pattern treatment printed on it. Because chameleon colour change may happen in a matter of seconds [3,14], chameleons were photographed immediately, after 1, 3 and then at 3 min intervals until 21 min. To better capture the chameleons’ initial response to the change in background colour, we conducted a second trial of the colour experiment by taking photographs at 0, 1, 3, 6 and 9 min. During trials, an effort was made to start with a different individual each time to reduce potential ordering effects, and the order of testing was pseudorandom. Trials were terminated if chameleons appeared distressed (see electronic supplementary material), were attempting to escape the arena, or when trials took longer than 40 min. Chameleons could not see human observers during the trials, except when the trapdoor (just large enough for the camera lens) was opened to take an image, the duration of which was minimized. For further details on chameleon husbandry, colour selection and pattern creation, see electronic supplementary material. The project was approved by the Exeter CEC ethics board.

### (b) Photographic capture and analysis

Images were captured using a full spectrum converted Nikon D7000 (Advanced Camera Services, Norfolk, UK), Coastal Optics 105 mm quartz ultraviolet (UV) lens (Coastal Optical Systems) and a UV and infrared (IR) blocking filter (Baader UV/IR Cut filter; transmitting between 400 and 700 nm). Two Zenith diffuse sheet grey standards of 8.6 and 95.8% reflectance were photographed in the same position and lighting conditions as the chameleons. The light was provided by two UV-emitting lamps (Zoo Med Reptisun 5.0 UVB T5 with starter unit) suspended at a fixed distance from the chameleon subject. Photographs were taken at an ISO of 120 with an F-stop of 8.

### (c) Predator vision model

We analysed the chameleon colour and pattern data using a model of peafowl (*Pavo cristatus*) vision, which has a violet-sensitive visual system [21]. The peafowl model was the closest representative available of the visual systems of some of the natural avian predators of chameleons, which include fiscal shrikes [3]. Although chameleons and many of their natural predators can see UV light, we did not focus on this aspect of their camouflage, although previous studies have [3,4] since it is practically very challenging to print backgrounds with UV reflectance. Additionally, UV images have much longer exposure times than visible images, and chameleons tended to move between visible and UV photos, making image alignment and measurement difficult.

### (d) Luminance and hue analysis

Chameleon colours were measured using the Multispectral Image Analysis Toolbox [22]. Within each image, a region of interest (ROI) was specified by drawing a polygon around the flank of the chameleon and a section of the background. A portion of the chameleon’s flank was selected based on the position of the chameleon to the camera. As chameleons were mobile, this portion varied slightly from image to image, but we avoided using the white marking found on the flank of flap-necked chameleons. Average peafowl cone-catch quanta were measured in each ROI, and the background was compared to the chameleon colours using the receptor noise limited model, providing ‘just noticeable differences’ for colour [23]. A trichromatic peafowl model was used, excluding UV [1,24]. For the luminance analysis, we used values obtained from the double cones, as double cones are thought to be responsible for achromatic vision in birds [16]. Average JNDs were calculated following Siddiqi *et al.* [25] using a Weber fraction of 0.05.

### (e) Pattern analysis

We tested chameleon responses to black and white, and green and yellow backgrounds of three different pattern sizes (see electronic supplementary materials for details of pattern creation). The pattern was measured from peafowl double cone images for each chameleon at successive time points. We analysed each ROI using a fast Fourier bandpass filtering approach [22,26]. This recorded the amount of ‘energy’ (the standard deviation luminance values of the filtered image) at each spatial scale (size of pattern) present within the experimental pattern being tested against (measured on a log scale with a multiplier of 1.414 beginning at 2 pixels). In this analysis, the ‘dominant spatial scale’ is the scale represented by the most energy in the ROI from the background treatment. We then compared the energy difference between the chameleons and the background at this scale over time to determine the extent to which the chameleons were pattern matching the background. The largest pattern size was 724 pixels, as this value represented the length of the smallest chameleon’s flank. Images were scaled to 18 pixels mm^−1^, and the average flank portion was 80 ± 5 mm in length.

Next, we compared the chameleon pattern spectrum to the background pattern spectrum to assess how they differed in pattern energy across spatial scales. This is known as the ‘pattern distribution difference’, where a value close to zero denotes no discernible difference between the chameleon and the background [27]. The ROI on each chameleon’s flank was compared to the three backgrounds of differing spatial frequency to discern whether chameleons more closely matched the pattern they were exposed to than the other two.

### (f) Statistical analyses

Statistical analyses were undertaken in R 4.2.1 [28]. For both luminance and hue experiments, we used linear mixed effects models (lmer) from the *lme4* package v. 1.1.30 [29] to measure the hue (comparative colour ratios of short wave, medium wave and long wave) and luminance of the chameleons in comparison to their backgrounds over time. These models included an interaction between time and background, e.g. lmer (chameleon colour approx. time × background colour + (1|ID)). Individual was used as a random effect, and for the colour experiments, trial number was an additional random effect. Full pairwise comparisons were computed using the *emmeans* package v. 1.11.0 [30]. The resulting *p*-values were adjusted for multiple testing using Benjamini–Hochberg correction from base R [28].

For the pattern experiments, we first tested whether the chameleons increased their expression of the background dominant spatial frequency over time using a linear mixed-effects model, with the individual as a random effect, e.g. lmer (pattern energy approx. time × treatment + (1|ID)). Finally, we used an ANOVA on the pattern distribution differences to test whether chameleons’ patterns more closely matched the spatial frequency they were exposed to than other spatial frequencies, running one ANOVA for green and yellow patterns and one for black and white.

## 3. Results

### (a) Hue matching

**Figure 1.**
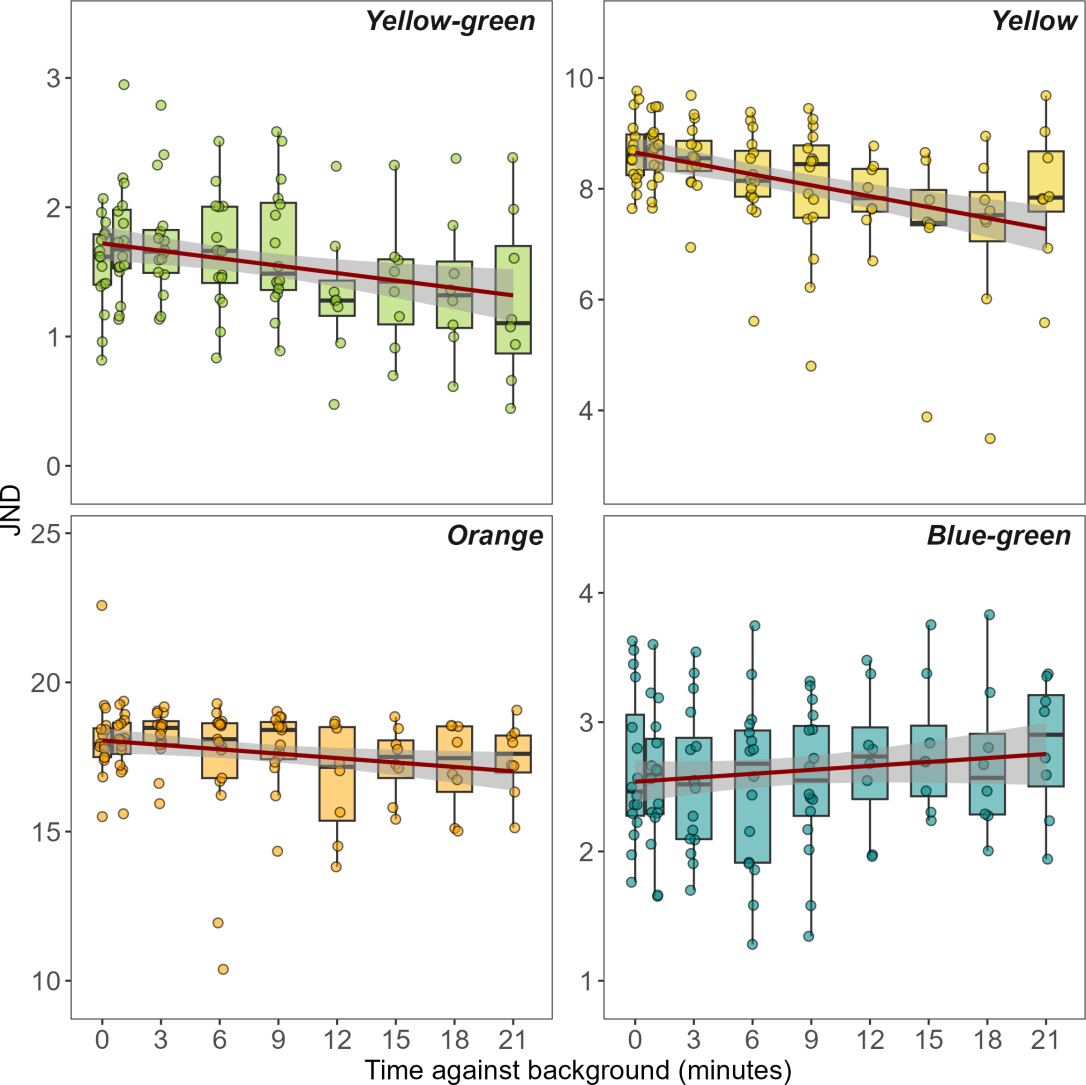
Colour discrimination (‘just noticeable difference’) values between chameleon flanks and the background arenas over time, calculated from the avian visual model. Values of <1 would make the chameleon flank indistinguishable in colour from the background. Lines represent smoothed conditional means with 95% confidence intervals, created using linear models applied through the geom_smooth function in the ggplot2 R package. Box widths at 0 and 1 min were reduced to prevent overlap.

Chameleons differed in the rate at which they changed colour depending on the background colour they were placed on (model comparison with interaction with time, *X*_2_ = 9.621_3_, *p* = 0.022, figure 1). Estimated slopes from the linear mixed-effects models are presented in table 1, and the results of all pairwise comparisons are in table 2. Chameleons changed colour fastest and improved colour matching most on the yellow background (*t* = −3.935, *p* < 0.001), and the reduction in the difference between chameleon and background appeared faster on yellow than both blue-green and yellow-green, although statistically borderline (yellow versus blue-green contrast; *t* = −2.517, *p* = 0.176, yellow-green versus yellow contrast; *t* = 2.571, *p* = 0.176). The rate of change did not differ meaningfully when comparing yellow versus orange (*t* = −0.896, *p* = 0.968) or yellow-green versus blue-green (*t* = −0.079, *p* = 0.9998). Chameleons increased their similarity to the orange background over time (*t* = −2.701, *p* = 0.014), and although this appeared to happen more quickly than on yellow-green or blue-green backgrounds, neither model suggested a significant effect (orange versus blue-green contrast; *t* = −1.666, *p* = 0.514, yellow-green versus orange contrast; *t* = 1.675, *p* = 0.514).

**Table 1.** Estimated slopes from the linear mixed-effects models showing changes in chameleon flank colour and brightness over time when placed on the background treatments. For colour experiments, negative estimates indicate increasing chameleon similarity to the background over time due to decreasing ‘just noticeable difference’ between chameleon and the background. For luminance experiments (grey, black and white), positive and negative estimates denote lightening and darkening over time, respectively. Asterisks represent levels of significance from the *p-*values: *p ≤ 0.05, **p ≤ 0.01, and ***p ≤ 0.001.

background	trend over time	s.e.	d.f.	lower CL	upper CL	*t*-ratio	*p*-value
yellow-green	−0.005	0.013	432.965	−0.031	0.021	−0.397	0.778
yellow	−0.052	0.013	432.965	−0.078	−0.026	−3.935	<0.001***
orange	−0.036	0.013	432.970	−0.061	−0.010	−2.701	0.014*
blue-green	−0.004	0.013	432.965	−0.030	0.022	−0.282	0.778
grey	0.001	0.002	210.000	−0.004	0.005	0.219	0.827
black	−0.010	0.002	210.004	−0.014	−0.005	−3.849	<0.001***
white	0.004	0.002	210.000	−0.001	0.009	1.545	0.186

**Table 2.** Results of the linear mixed-effects model examining the effect of time on chameleon flank similarity to the background during hue experiments. Each model sets a different background as the reference, and all model pairs were tested. For hue experiments, changes in the ‘just noticeable difference’ between the chameleon and the background are measured over time. For luminance experiments (grey, black and white), estimates represent a change in the double cone values, signifying brightness. Significant interactions indicate that the slope over time differs between the experimental background and the reference background. In these cases, the degree of similarity between the chameleon and the background changes at different rates between the test background and reference. Asterisks represent levels of significance from the *p-*values: *p ≤ 0.05, **p ≤ 0.01, and ***p ≤ 0.001.

contrast (test background versus reference)	estimate	s.e.	d.f.	*t*-ratio	*p*-value
yellow-green versus yellow	0.047	0.018	432.000	2.571	0.176
yellow-green versus orange	0.030	0.018	432.001	1.675	0.514
yellow-green versus blue-green	−0.002	0.019	432.697	−0.079	0.9998
yellow versus orange	−0.016	0.018	432.001	−0.896	0.968
yellow versus blue-green	−0.048	0.019	432.697	−2.517	0.176
orange versus blue-green	−0.032	0.019	432.693	−1.666	0.514
grey versus black	0.010	0.003	210.002	2.975	0.014*
grey versus white	−0.003	0.003	210.000	−0.984	0.588
black versus white	−0.013	0.004	210.002	−3.816	0.002*

### (b) Luminance matching

**Figure 2.**
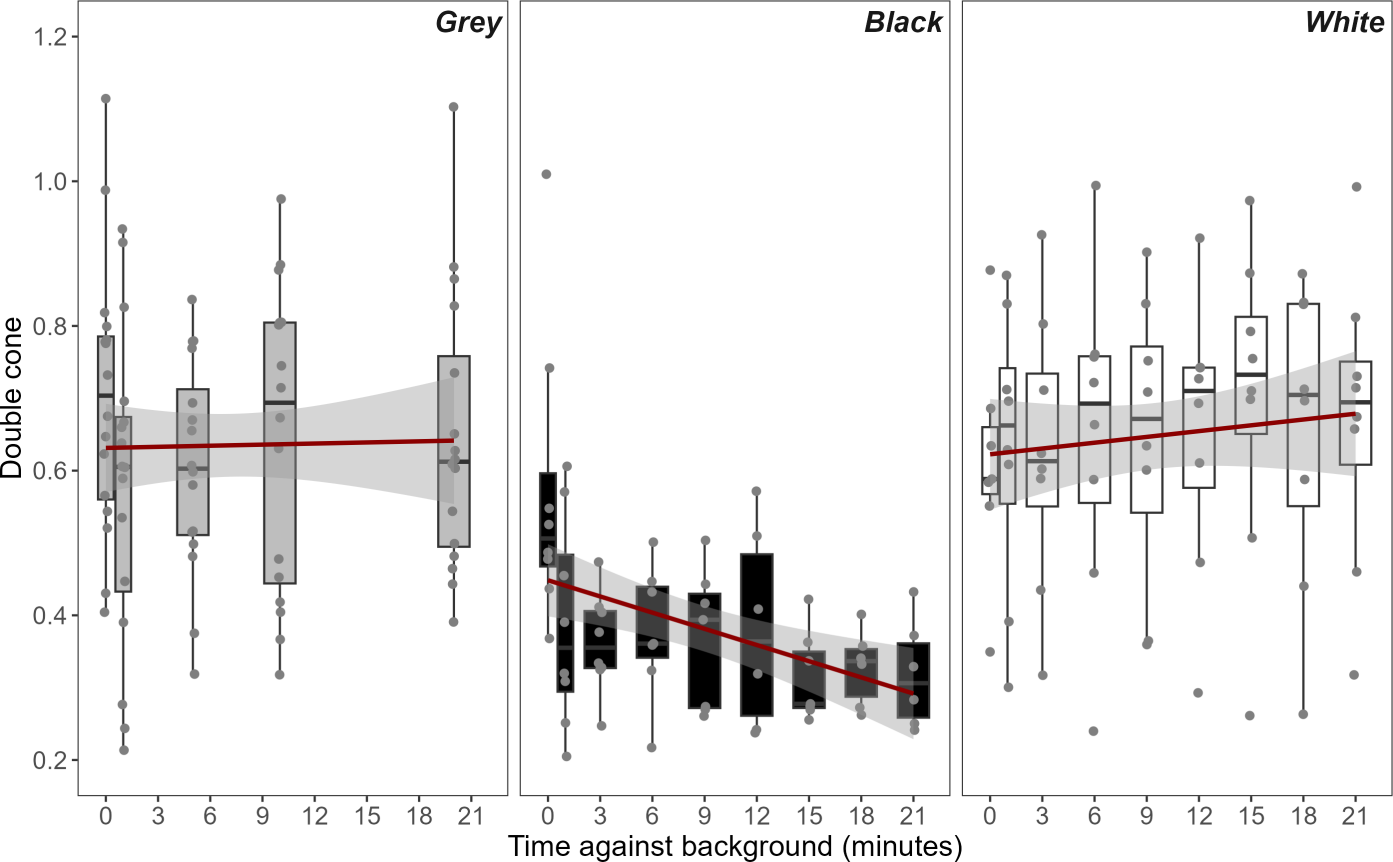
Double cone values of chameleon flanks over time when placed on either grey, black or white arenas, calculated from the avian visual model. Double cones mediate achromatic discrimination in birds, and a reduction or increase in the double cone value denotes the darkening and lightening of the subject, respectively [16]. Lines represent smoothed conditional means with 95% confidence intervals, created using linear models applied through the geom_smooth function in the ggplot2 R package. Box widths at 0 and 1 min were reduced to prevent overlap.

When placed on a black background, luminance (double cone) values from the flanks of chameleons became significantly darker over time (*t* = −3.849, *p* ≤ 0.001, mean luminance decrease of 23.3%, table 1, figure 1). The darkening was also more pronounced on the black than on the grey control background (*t* = 2.975, *p* = 0.014, table 2, figure 2) or white background (*t* = −3.816, *p* = 0.002). When exposed to the white treatment, chameleon luminance values became lighter over time, although this was not statistically significant (*t* = 1.545, *p* = 0.186, mean luminance increase of 20.7%, table 2, figure 2). Chameleons appeared to lighten more quickly on white than while on the grey control background, although this was not significant (*t* = −0.984, *p* = 0.588).

### (c) Pattern matching

Chameleons were unable to increase their expression of the dominant pattern size on any of the different spatial frequencies on different patterned backgrounds nor over time (electronic supplementary material, table S1). Nor did chameleons match the spatial frequency of the treatment they were exposed to more closely than either of the other two treatments based on the pattern distribution differences. This was true for the black and white patterned treatments (F_1, 70_ = 0.045, *p* = 0.833) and green and yellow patterned treatments (F_1, 70_ = 0.298, *p* = 0.587). It is worth noting that chameleons had a lower pattern distribution difference when compared with the green and yellow backgrounds (range 299–736) than black and white backgrounds (range 2965–3795), suggesting they are significantly better camouflaged in yellow and green environments than black and white ones when viewed by the avian predator.

## 4. Discussion

Our results demonstrate facultative crypsis in flap-necked chameleons in the absence of a predatory stimulus. Colour change occurred against black, orange and yellow backgrounds, but against white, it was statistically borderline. Chameleons did not change significantly to match the blue-green or yellow-green backgrounds, although they were an excellent colour match to these backgrounds already. There was no discernible alteration in their appearance to suggest an effort to match patterned backgrounds.

The ability to change colour has likely evolved as a means for chameleons to maintain their camouflage as they move through heterogeneous patches of habitat [31]. Flap-necked chameleons are found in areas of mixed vegetation with distinct wet and dry seasons. These comprise grasses, bushes and trees, and chameleons also spend time on the ground [20], so an ability to alter their appearance in accordance with their perch or substrate type would likely convey a survival advantage [11,20]. Indeed, ground and vegetation colour influence variation in the colour of chameleon flanks between populations of *C. dilepis* in South Africa [18], but whether this is due to individual colour change or interpopulation variation is unclear. While it has been suggested that flap-necked chameleons exhibit different coloration between wet and dry seasons to match green or brown foliage [20], our study found evidence for physiological colour change over a period of minutes, allowing chameleons to swiftly increase their similarity to the background.

Despite their famed abilities, it is unlikely that chameleons can match a wide variety of background colours. Common chameleons turn brown or green depending on the bush they are inhabiting, but do not match off-white, yellow or blue backgrounds [7]. Similarly, rock gobies (*Gobius paganellus*) can only match certain colours, and they compensate behaviourally by choosing substrates they can match [32]. In our study, yellow, and to a lesser extent orange, may be the most biologically relevant colours for flap-necked chameleons to match that we tested. It is also possible that because some of the colours were already very close to the chameleon’s colour, they did not elicit a significant colour change. The discrimination values for blue-green and yellow-green were both less than three at the beginning of trials (figure 1). Future studies should carefully consider relevant colours for these species to try and replicate, and since there is evidence for changes to brown in both flap-necked and common chameleons, it would represent a logical starting point for further investigation. In addition, while the importance of colour change for communication is strongly supported, investigating when colour change is prioritized for communication, crypsis or thermoregulation, or whether colour and patterning may change over a range of more complex backgrounds will continue to prove fruitful. While we did not find evidence of pattern-matching in flap-necked chameleons, it is possible that our experimental treatments did not elicit this behaviour or that pattern changes take place over longer time periods than our 21 min trials.

Chameleons are energy-conservative animals [5,33], and it is widely assumed that there are energetic costs associated with colour change [34]. High physiological effort may, therefore, be an explanation for chameleons not constantly matching their background as closely as possible [4]. Other colour-changing animals will minimize energy expenditure by avoiding colour change unless necessary [35], and it may often be more cost-effective for chameleons to move rather than match a background. Future work should investigate the rate-dependent metabolic costs of colour change [35,36], and how the rates of colour change link to temporal perception thresholds in predators and conspecifics. Our documentation of flap-necked chameleons using facultative crypsis to match both hue and brightness adds to our understanding of the ecology of this charismatic group of lizards and highlights the remaining gaps in our knowledge of colour-changing terrestrial vertebrates.

## Data Availability

All data and code [37] and supplementary materials [38] are available online from the figshare repository.
